# Physical Activity, Nutritional Status, and Autonomic Nervous System Activity in Healthy Young Adults with Higher Levels of Depressive Symptoms and Matched Controls without Depressive Symptoms: A Cross-Sectional Study

**DOI:** 10.3390/nu12030690

**Published:** 2020-03-04

**Authors:** Natsuki Tsujita, Yasunori Akamatsu, Márcio Makoto Nishida, Tatsuya Hayashi, Toshio Moritani

**Affiliations:** 1 Graduate School of Human and Environmental Studies, Kyoto University, Yoshida Nihonmatsu-cho, Sakyo-ku, Kyoto 606–8501, Japan; natsuki.morita823@gmail.com (N.T.); akamatsu.yasunori@gmail.com (Y.A.); tatsuya@kuhp.kyoto-u.ac.jp (T.H.); 2 Graduate School of Medicine, Kyoto University, 53 Kawahara-cho, Shogoin, Sakyo-ku, Kyoto 606–8397, Japan; marcio.nishida@gmail.com; 3 Department of Health and Sports Science, Kyoto Sangyo University, Kamo-motoyama, Kita-ku, Kyoto 606–8555, Japan

**Keywords:** depression, diet, heart rate variability, lifestyle, mental health, physical activity, tryptophan, vitamin B_6_

## Abstract

The aim of the present study was to comprehensively investigate physical activity (PA), nutritional status, and autonomic nervous system (ANS) activity in healthy young adults with higher levels of depressive symptoms and in sex- and age-matched controls without depressive symptoms. We recruited 35 healthy young adults with higher levels of depressive symptoms (DEP group) and 35 controls (CON group). Measurement items were daily number of steps, the duration and amount of PA ≥3 metabolic equivalents (METs), exercise habits, the consumption of tryptophan (TRP) and/or vitamin B_6_-rich foods, plasma levels of total TRP and vitamin B_6_ levels, and ANS activity. The DEP group had fewer daily steps, as well as duration and amount of PA ≥3 METs, than the CON group, while there was no difference in exercise habits. The intake frequency of TRP and/or vitamin B_6_-rich foods and plasma vitamin B_6_ levels of the DEP group were rather higher than those in the control group. Plasma TRP levels and ANS activity were comparable in the two groups. Our findings suggest that a decline in overall PA, including daily steps as well as duration and amount of moderate-to-vigorous-intensity PA, could be associated with higher levels of depressive symptoms in healthy young adults. Their dietary intake of TRP and/or vitamin B_6_-rich foods was adequate, and there was no ANS activity dysfunction.

## 1. Introduction

The number of people with depression is continuously increasing, with an estimated 300 million individuals worldwide affected currently by depression [1]. There are a lot of previous studies on depression; however, most of them are about depressed patients, with few studies conducted to date on healthy subjects in the pre-stage of depression. In Japan, it was reported that the 12-month incidence of major depressive episodes was approximately 20% among first-year university students [2]. There is also evidence suggesting that depression may have a more negative impact on adolescents than adults [3], and depressive symptoms early in life are associated with an increased risk of affective disorders during adulthood [4]. Despite this, 16- to 24-year-olds are the least likely age group to have adequate access to mental health services [5]; therefore, it is important to prevent the onset of depressive symptoms at an early stage before the appearance of clinical depression.

Previous studies have consistently shown that physical activity (PA) alleviates depressive symptoms [6]. Low PA levels are associated with depression onset [7], while higher PA levels are consistently associated with lower odds of developing depression in the future [8].

Nutritional status, particularly with regard to tryptophan (TRP) and vitamin B_6_ levels, may also have an important effect on depressive symptoms. TRP is a precursor of serotonin. TRP depletion may lead to mood disorders [9]. Vitamin B_6_ acts as a coenzyme in the TRP–serotonin metabolic pathway [10], and low levels of vitamin B_6_ have previously been associated with depressive symptoms [11].

Autonomic nervous system (ANS) activity may also be connected with depressive symptoms. In earlier studies, patients with depression exhibited increased sympathetic activity and/or decreased parasympathetic activity [12,13]. However, conflicting results regarding the relationship between depression and ANS activity have been reported. This variation may be explained by several reasons. First, ANS activity is influenced by a number of different physiological factors, such as sex, age [14], medication [15], PA [16], and food intake [17]. Differences in methodology can also influence ANS activity. Our heart rate variability (HRV) power spectral analysis is a well-accepted, useful, and noninvasive method for assessing ANS activity. In previous studies, we performed HRV power spectral analysis for comprehensive quantitative and qualitative evaluations of neuroautonomic functions under various physiological conditions [16,17,18,19]. It has been found that HRV is reduced with increases in depression severity [20]. We recently showed that the return to a work program for three months might improve parasympathetic activity in workers on sick leave due to major depression or bipolar disorder [21]. Therefore, in order to obtain reliable ANS activity data, it is necessary to use HRV power spectral analysis and to investigate PA and food intake at the same time for age- and sex-matched healthy young adults without a diagnosis of depression.

Many studies have investigated PA, nutritional status, and ANS activity, respectively, in depressed patients, while the examination of these characteristics simultaneously in healthy subjects in the pre-stage of depression is rarer. Identifying the characteristics of healthy young adults in the pre-stage of depression may facilitate the development of preventive interventions and help to reduce the incidence of depression. The aim of the present study; therefore, was to comprehensively investigate PA, nutritional status, and ANS activity among healthy young adults in the pre-stage of depression and sex- and age-matched nondepressive controls and to elucidate any differences between them. We hypothesized that healthy young adults in the pre-stage of depression would display lower levels of PA, a lack of TRP and/or vitamin B_6_-rich foods in their diet, and decreased ANS activity as compared with nondepressive controls. In this study, depressive symptoms were measured only using the Center for Epidemiological Studies Depression Scale (CES-D), and subjects did not participate in a depression diagnostic interview; therefore, we use the term “healthy individuals with higher levels of depressive symptoms” to refer to healthy young adults in the pre-stage of depression and “healthy individuals without depressive symptoms” to indicate nondepressive controls.

## 2. Materials and Methods

### 2.1. Subjects

We recruited 140 students from a Japanese university who completed the CES-D questionnaire. Among them, 61 students (28 males and 33 females) demonstrated higher levels of depressive symptoms; as a result, 35 of these (14 males and 21 females) volunteered to participate in the study in the higher levels of depressive symptoms (DEP) group. Separately, 35 subjects matched for age (within a two-year range) and sex were selected in order from among the remaining 79 students (32 males and 47 females) with the lowest scores and assigned to the control (CON) group. Thus, 35 students with higher levels of depressive symptoms and 35 students without depressive symptoms aged 18 to 26 years were enrolled in this study. All subjects were in good physical health and had no personal or family history of hypertension, cardiovascular disease, diabetes mellitus, or other endocrine diseases; further, none of the subjects were taking medication. The subjects’ demographic characteristics are summarized in Table 1. Written informed consent was obtained from all the subjects prior to their enrollment in accordance with the Declaration of Helsinki. The study was approved by the Ethical Committee of the Graduate School of Human and Environmental Studies at Kyoto University (25-H-23). 

### 2.2. Depressive Symptoms

Depressive symptoms were assessed using the Japanese version [22] of the CES-D, an instrument used to determine depressive states among individuals in the general population [23]. The instrument contains 20 questions related to emotions, such as loneliness and sadness. Subjects were required to indicate how often they had experienced such emotions in the past week. Each question was assigned a score on a scale of zero to three points; thus, the maximum possible total score for the 20 questions was 60 points. A score of ≥16 was considered indicative of higher levels of depressive symptoms.

### 2.3. Exercise Habits

Subjects were asked how frequently they participated in sports activities lasting more than 30 min. Sports activities included both club-based activities and personal activities such as running.

### 2.4. Nutritional Status Assessment

Nutritional status was assessed using a food frequency questionnaire based on food groups (FFQg). The validity of the FFQg was assessed by comparing dietary records of 66 subjects aged 19 to 60 years for a week [24]. The instrument is a four-page questionnaire covering the frequency of intake of certain kinds of foods during a week, classified into 29 food groups (e.g., grains, seafood, and seaweed) and 10 modes of cooking (e.g., boiling and frying). For example, one question in the meat category was “how much meat do you eat in a week and how often do you eat it?” For comparison, pictures of two or three thin slices of meat or four slices of ham are shown as normal serving sizes. Subjects indicated their intake frequency over a week as “a lot,” “normal,” “a little,” or “none” and reported the number of times they consumed meat as a part of breakfast, lunch, and supper. Nutritional values were calculated using computer-assisted procedures (Excel EIYOKUN, version 5.0, KENPAKUSYA Co., Tokyo, Japan) based on the Japanese food consumption table [25].

### 2.5. Measurement of PA

All subjects were equipped with activity trackers (Activity Style Pro HJA-350IT; Omron Healthcare, Kyoto, Japan) for three days, which included two weekdays and one holiday. They were instructed to maintain their normal PA during the study and asked to wear their activity trackers all waking hours, exclusive of any time spent bathing or in water. The activity tracker contains a three-dimensional accelerometer that allows it to measure not only the number of steps taken by the subjects but also PA intensity expressed in metabolic equivalents (METs). METs have been used as an indication of PA intensity [26], and a MET is defined as the resting metabolic rate; that is, the amount of oxygen consumed at rest, sitting quietly in a chair, approximately 3.5 mL/kg/min. Energy consumption was easily calculated by using METs as METs × body weight × time in hours. For example, normal walking is about three METs; therefore, when a 50-kg person walks for one hour, their energy consumption is approximately 150 kcal. As an indicator of PA intensity, the average daily duration and amount of activity at an intensity of ≥3 METs were recorded for each subject. The daily amount of activity was expressed as METs h/day (calculated as sum of METs × time in hours). All data are blind to subjects while they are wearing the device. Published guidelines were followed to identify and remove days with incomplete invalid accelerometer wear time [27]. A valid day was defined as data with >10 h per day of valid wear time. Wear time was defined by subtracting nonwear time from 24 h. Nonwear time was defined as an interval of at least 60 consecutive minutes of 0 counts, with allowance for up to 2 min of up to 100 counts.

### 2.6. Consumption of TRP and/or Vitamin B_6_-Rich Food

Table 2 shows the 30 foods considered in this study and their TRP and vitamin B_6_ content as provided in the Standard Tables of Food Composition in Japan [25]. The foods were chosen from across the various food groups. Most typically contain more TRP and/or vitamin B_6_ per 100 g than other foods; however, the volume and intake of particular food items were also taken into consideration while identifying dietary sources of TRP and vitamin B_6_. For example, rice contains 83 mg TRP/100 mg as compared with fish or meat containing 200–300 mg/100 g; however, since rice is a staple food consumed in large quantities, it was included in the list. Conversely, pistachios or sunflower seeds were excluded. For each food on the list, study subjects were asked to indicate their frequency of consumption from the following four response options: “almost every day,” “once in two days,” “twice in a week,” and “almost never.”

### 2.7. Blood Investigations

Blood samples to measure the subjects’ total TRP and total vitamin B_6_ levels were collected in vacuum tubes (Venoject II Vacuum Tube; Terumo Co., Tokyo, Japan) and centrifuged at 3000 × g for 15 min at 4°C. The plasma samples were then stored at −20°C until they were necessary for further processing. The total TRP and vitamin B_6_ levels were determined with the sandwich enzyme-linked immunosorbent assay method using a TRP immunoassay kit (Abnova, Taipei, Taiwan) and vitamin B_6_ immunoassay kit (Cloud-Clone Corp., Houston, TX, USA), respectively. All of the assays were performed in duplicate as recommended by the manufacturers. The intra- and inter-assay coefficients of variation were both below 10%.

### 2.8. Measurement of ANS Activity

The interval between heartbeats (the R–R interval) fluctuates beat by beat. HRV measures the fluctuation around the mean heart rate, which reflects the cardiorespiratory control system. HRV is a valuable marker for the investigation of sympathetic and parasympathetic autonomic activity, as well as sympathovagal balance [28]. The electrocardiogram (ECG) R–R interval is determined by the net effect of the sympathetic and the parasympathetic inputs. HRV can be evaluated by analyzing R–R intervals using a computer system (HTBasic, TransEra, Utah, USA), which indicates ANS activity. The ECG signal was amplified (BBA-8321; Bio-Tex, Kyoto, Japan) and digitized via a 13-bit analog-to-digital converter (Daq AD 132; Elan, UK) at a sampling rate of 1024 Hz. The digitized ECG signals were differentiated, and the resultant ECG QRS spikes and intervals of the impulses (R–R intervals) were stored sequentially on a hard disk for later analyses. Before R–R spectral analysis was performed, the stored R–R interval data were displayed and aligned sequentially to obtain equally spaced samples with an effective sampling frequency of 2 Hz [29] and displayed on a computer screen for visual inspection. Next, the direct current component and linear trend were completely eliminated by digital filtering for the bandpass process between 0.03 and 0.5 Hz. The root mean square value of the R–R interval was calculated as representing the average amplitude. After passing through the Hamming-type data window, power spectral analysis by means of a fast Fourier transform was performed on a consecutive 256-s time series of R–R interval data obtained during the test. Generally, the high–frequency (HF) components of the power spectral analysis (0.15–0.5 Hz) of HRV are almost entirely associated with vagal nerve activity, whereas low–frequency (LF) components (0.03–0.15 Hz) may be mediated by both vagal and sympathetic activity [30]. Further, the total power (0.03–0.5 Hz) reflects the overall ANS activity. Due to considerable interindividual variability of basal spectral absolute values, the spectral powers were logarithmically transformed prior to statistical analysis.

### 2.9. Experimental Procedures

All subjects were instructed to avoid consuming any food or beverage containing alcohol or caffeine after 10:00 p.m. on the day preceding the study. On the day of the test, subjects arrived at the laboratory at 8:00 a.m. The room was temperature-controlled (25 °C), quiet, and comfortable, with minimal arousal stimuli. Body mass and percentage body fat were determined using a bioelectrical impedance analyzer (Model BC118-D; Tanita Corp., Tokyo, Japan). The subjects were then fitted with CM5 lead ECG electrodes and asked to rest for at least 15 min before beginning the experiment. After the resting period, the ECG signals were continuously recorded for five minutes while each subject remained seated in a chair. During the ECG recording, the subjects breathed in synchrony with a metronome at 15 times/min (0.25 Hz) to ensure that respiratory-linked heart rate variations did not overlap with LF heart rate fluctuations (0.03–0.15 Hz) from other sources [30]. Finally, blood samples to measure plasma TRP and vitamin B_6_ levels were obtained by a doctor. During the waiting period, subjects filled out the FFQg and other questionnaires about their exercise habits and the consumption of food items rich in TRP and vitamin B_6_. After the test day, the subjects were equipped with activity trackers for three days, which included two weekdays and one holiday.

### 2.10. Statistical Analysis

All data are presented as mean ± SD, and between-group differences were assessed using the Student’s unpaired *t*-test, with the exception of the intake frequencies of TRP and vitamin B_6_-rich diets. It was presented as the number and percentage of each response and assessed using the chi-square test. If any cell had a frequency < 5, Fisher’s exact test was used. Pearson’s coefficient of correlation was used for investigation of relationship between CES-D scores and other factors. All statistical analyses were performed using SPSS software version 21.0 (IBM Corp., Armonk, NY, USA). A *p*-value of less than 0.05 indicated a statistically significant between-group difference.

A sample size calculation was performed for the plasma concentration of TRP as the main outcome. The sample size was estimated based on a previous report [31]. A sample size of 27 participants per group was found to be required to detect a between-group difference of 1.43 µg/mL in the plasma concentration of TRP (power = 0.8; alpha = 0.05) G*Power, version 3.1.9 [32]). This predicted difference equates to a large effect size of ≥ 0.8. To account for an expected 10% dropout rate, a minimum sample size of 30 participants was thus required per group.

## 3. Results

### 3.1. Physical Characteristics

The physical characteristics of the DEP and CON groups are summarized in Table 1. No significant between-group differences were observed with respect to body mass, body mass index, percentage of body fat, amount of exercise, or resting heart rate. There were 11 subjects who met the Japanese exercise guidelines [33] (exercise for >30 min at least twice a week) in the CON group and 12 who met these guidelines in the DEP group. The estimated energy intake in the DEP and CON groups were nearly identical. 

### 3.2. PA 

**Figure 1:**
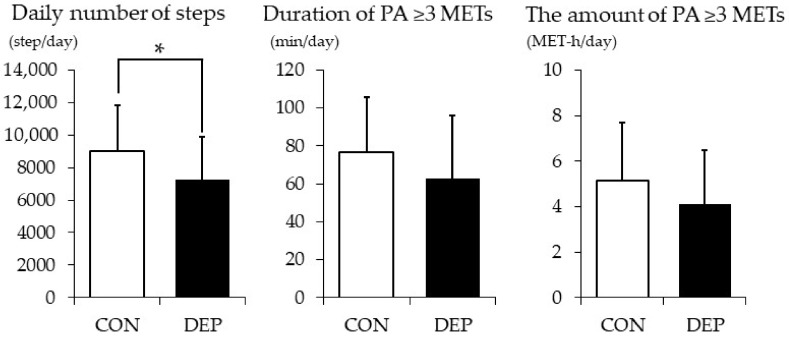
Daily number of steps, duration, and the amount of PA ≥ 3 METs. Values are presented as means ± SD. CON: control group; DEP: higher levels of depressive symptoms group, PA: physical activity, MET: metabolic equivalent. * *p* < 0.05.

Figure 1 shows the mean daily numbers of steps taken respectively by subjects in the two groups and the mean duration and the amount of PA ≥3 METs. The DEP group took significantly fewer steps per day as compared with the CON group (7232 ± 2664 vs. 8983 ± 2859 vs. steps/day, respectively; *t* score = 2.63, Cohen’s d = −0.63, *p* = 0.011). The mean duration (63.0 ± 33.0 vs. 76.7 ± 29.3 min/day, respectively) and amount (4.12 ± 2.35 vs. 5.14 ± 2.55 MET-h/day, respectively) of PA ≥3 METs in the DEP group were less than in the CON group, though the differences were not statistically significant (*t* score = 1.82, Cohen’s d = −0.44, *p* = 0.073 and *t* score = 1.71, Cohen’s d = −0.41, *p* = 0.091, respectively).

### 3.3. Consumption of TRP and/or Vitamin B_6_-Rich Food

Table 2 presents the intake frequency of TRP and vitamin B_6_-rich foods. Contrary to expectation, the DEP group consumed significantly more bonito and mackerel in comparison with the CON group (*p* < 0.01). The DEP group also ate significantly more pacific saury, horse mackerel, beef liver, and chicken liver than did the CON group (*p* < 0.05). 

### 3.4. Plasma TRP and Vitamin B_6_ Levels

**Figure 2:**
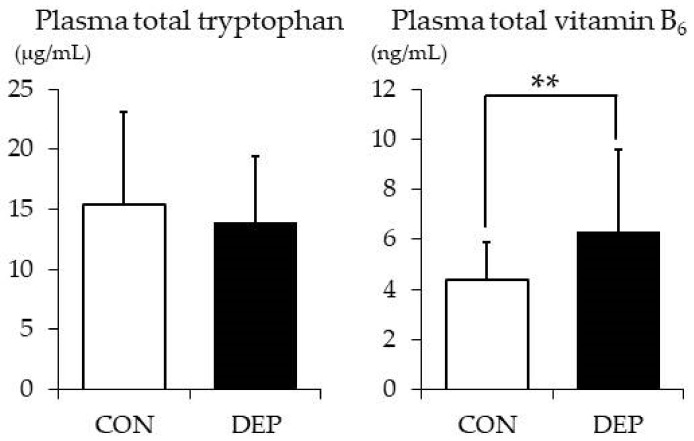
Plasma total tryptophan and vitamin B_6_ levels. Values are presented as means ± SD. CON: control group and DEP: higher levels of depressive symptoms group. ** *p* < 0.01.

Plasma total TRP and vitamin B_6_ levels in the two groups are shown in Figure 2. The mean plasma total TRP level was comparable among the two groups (CON vs. DEP: 15.4 ± 7.7 vs. 14.0 ± 5.4 μg/mL; *t* value = 0.85, Cohen’s d = −0.08, *p* = 0.398). However, in contrast, the mean plasma vitamin B_6_ level in the DEP group was significantly higher than that in the CON group (6.3 ± 3.3 vs. 4.4 ± 1.5 ng/mL, respectively; *t* value = −3.04, Cohen’s d = −0.95, *p* = 0.004).

### 3.5. ANS activity 

Table 3 presents the logarithmic values of total power, LF power, HF power, and LF/HF. No significant between-group differences were observed with respect to any of the HRV indices, although total power, LF power, and HF power in the DEP group were higher than in the CON group. In comparison, LF/HF was nearly identical in the two groups.

### 3.6. Coefficient of Correlation

The CES-D scores showed a significant negative correlation with steps, duration, and the amount of PA ≥3 METs (r = −0.358, *p* = 0.003; r = −0.296, *p* = 0.014; and r = −0.286, *p* = 0.017, respectively). On the other hand, a positive correlation was found between the CES-D scores and plasma vitamin B_6_ level (r = 0.288, *p* = 0.021). With regard to plasma TRP level and ANS activity, no correlation with CES-D score was found (plasma TRP level: r = −0.106, *p* = 0.388; total power: r = 0.134, *p* = 0.277; LF power: r = 0.111, *p* = 0.368; and HF power: r = 0.141, *p* = 0.251, respectively).

## 4. Discussion

The main finding of this study was that healthy young adults with higher levels of depressive symptoms had fewer daily steps as well, as the duration and amount of PA ≥3 METs, than their control group counterparts, despite showing no difference in exercise habits. In addition, the intake frequency of TRP and/or vitamin B_6_-rich foods and plasma vitamin B_6_ levels of the DEP group were rather higher than those in the control group. Plasma TRP levels and ANS activity were comparable in the two groups.

The mean daily step count provided an objective measure of significantly lower PA in the DEP group, which is consistent with previous study results. Yoshiuchi et al. studied a year-averaged step count, recorded by accelerometer, in 500 elderly people for a period of >15 years; the study revealed that older adults who take at least 4000 steps/day were less likely to experience depressive symptoms [26]. Elsewhere, McKercher et al. investigated step counts of 1995 young adults aged 26 to 36 years using a pedometer; among the female subjects, PA of ≥ 7500 steps/day was associated with a 50% lower prevalence of depression when compared with a step count of < 5000 steps/day [34]. Additionally, duration and the amount of PA ≥3 METs, which is an indicator of moderate- and vigorous-intensity PA, tended to be lower in the DEP group. These factors negatively correlated with depressive symptoms, as well as daily steps. Considering that more subjects in the DEP group than in the CON group met the Japanese exercise guidelines (exercise for >30 min at least twice a week), PA intensity may be associated with higher levels of depressive symptoms in young adults. One plausible reason for this is energy consumption is relevant. Paffenbarger et al. reported that a trend of reduced risk of depression with increased levels of energy expenditure was observed in American college men [35]. Dunn et al. compared two exercise treatments that varied in terms of total energy expenditure and reported that greater energy expenditure was associated with more significant reductions in depressive symptoms [36]. Given that a steep decline in PA occurs during late adolescence and young adulthood [37], decline in overall PA, including daily steps as well as duration and amount of moderate-to-vigorous-intensity PA, may lead to a reduction in energy expenditure, which could be associated with higher levels of depressive symptoms in healthy young adults. 

There was no difference between the DEP and the CON groups in terms of estimated dietary energy intake. When considering the reported TRP and/or vitamin B_6_-rich foods, subjects in the DEP group consumed more fish than did those in the CON group, contrary to our expectation. These results differ from most previous studies, in which fish consumption was shown to protect against depression [38]. One plausible explanation is that the protein also includes different amino acids other than TRP. TRP converts to serotonin in the presence of vitamin B_6_ [10]. TRP’s pathway to the brain is, however, shared by other large neutral amino acids (LNAAs), which compete with TRP for transport across the blood–brain barrier [39]. TRP dietary intake from proteins is quite small (< 1 g/day) [40]. Thus, TRP and/or vitamin B_6_-rich foods, especially fish, may increase LNAAs in addition to TRP, which may lead to competition with the TRP and may not result in serotonin synthesis. This explanation is supported by the nearly identical plasma TRP levels in the two groups. Decreased plasma TRP levels were found elsewhere in depressed patients [41], while it is reported that plasma TRP is significantly lower in patients with somatization, even in the absence of depression [42]. It has also been suggested that immunoinflammatory responses in depression lowered plasma TRP, while TRP depletion techniques induced physiosomatic symptoms in healthy volunteers [43]. That is, decreased plasma TRP is more closely related to somatization than depressive symptoms. In this study, subjects had higher levels of depressive symptoms but no physiosomatic symptoms; therefore, their plasma TRP levels did not differ from those of the control group.

The plasma vitamin B_6_ level in the DEP group was significantly higher than that in the CON group and showed a positive correlation with depressive symptoms, while previous studies reported that low levels of vitamin B_6_ are associated with depressive symptoms [11]. It is possible that when one’s depressive symptoms become higher, they feel a stronger urge to eat TRP and/or vitamin B_6_-rich foods to synthesize serotonin. As a result, TRP and vitamin B_6_ become sufficiently present in the blood; however, it is thought that serotonin synthesis does not occur well in the brain due to competition with TRP and other LNAAs. Our previous study showed that TRP, vitamin B_6_, and nicotinamide-containing supplements loading between meals, when the competitive influences of other LNAAs are small, can quickly improve depressed moods in quite low doses in young adults with severe subclinical depression, while no such effect was found in taking the same supplements after meals [44]. That is, unlike depressed patients, healthy young adults with higher levels of depressive symptoms showed adequate dietary intakes of TRP and vitamin B_6_, and their blood levels did not decrease.

In this study, no difference in ANS activity was observed between the two groups. Some previous studies reported reduced HRV in depressed patients, although the control groups were not matched for age and sex [12,13]. Conversely, some studies that employed sex- and age-matched controls revealed no difference in HRV was present between depressed patients and matched controls [45,46]; this finding suggests that ANS activity is influenced more by physiological factors than by depression itself. Considering healthy subjects, no significant baseline difference was found in HF between the high and low depressed mood groups [47]. In this study, subjects had higher levels of depressive symptoms, but they were not clinically depressed; hence, ANS activity dysfunction may have not occurred. Recently, it was reported that depressed adolescents presented reduced complex cardiovagal control during physiological and mental stress as compared with healthy controls [48]. It is implied blunted ANS activity was present in response to physiological stimulation in even healthy young adults with higher levels of depressive symptoms; hence, further research is needed to provide more definitive evidence in this regard.

The present study revealed that health young adults with higher levels of depressive symptoms had fewer daily steps, as well as duration and amount of moderate-to-vigorous-intensity PA, than those in the control group. Their dietary intake of TRP and/or vitamin B_6_-rich foods was adequate, and there was no ANS activity dysfunction. From these results, it was found that healthy young adults with higher levels of depressive symptoms differ from depressed patients even though they have higher levels of depressive symptoms. Low levels of TRP and/or vitamin B_6_ and ANS activity dysfunction reported in depressed patients may be due to medication or clinical depressive symptoms. 

The present study had some limitations that should be mentioned. First, the exercise habit questionnaire was self-developed; its reliability and validity have not been verified elsewhere. However, the questionnaire results were consistent with our objective data, which showed no significant between-group differences with respect to duration and the amount of PA ≥3 METs. Second, activity trackers were equipped for only three days. Third, the questionnaire about the consumption of TRP and/or vitamin B_6_-rich foods was also self-developed. However, the results of blood investigations were consistent with the higher dietary intakes of TRP and/or vitamin B_6_-rich foods in the depressive group. Finally, this was a cross-sectional study, which does not permit any causal inferences. 

## 5. Conclusions

Our findings suggest that a decline in overall PA, including daily steps as well as duration and amount of moderate-to-vigorous-intensity PA, could be associated with higher levels of depressive symptoms in healthy young adults. Their dietary intake of TRP and/or vitamin B_6_-rich foods was adequate, and there was no ANS activity dysfunction.

## Figures and Tables

**Table 1:** Descriptive characteristics of subjects.

	CON		DEP		*t* Value	Cohen’s d	*p* Value
	*n* = 35		*n* = 35				
Age (years)	20.3	(1.6)	20.5	(2.1)	−0.575	−0.11	0.567
Body mass (kg)	54.3	(9.2)	54.5	(10.8)	−0.050	0.02	0.960
BMI (kg/m^2^)	20.3	(3.2)	20.0	(2.1)	0.484	−0.11	0.630
Body fat (%)	21.8	(6.7)	22.1	(6.6)	−0.210	0.05	0.834
Exercise (times/week)	1.2	(1.8)	1.5	(2.2)	−0.685	0.16	0.496
Resting heart rate (bpm)	69.8	(9.6)	69.3	(9.6)	0.224	−0.06	0.823
Energy intake (kcal/day)	1815	(433)	1839	(452)	−0.251	0.06	0.820
CES-D scores	3.9	(2.2)	25.1	(7.4)	−16.125	3.86	**0.000**

Values are presented as means (SD). CON: control group; DEP: higher levels of depressive symptoms group; and CES-D: Center for Epidemiologic Studies Depression Scale. Significant *p* values are in bold type.

**Table 2:** Weekly frequency of intake of food items rich in tryptophan and vitamin B_6_ and their contents per 100 g of food.

	Tryptophan Contents (mg)	Vitamin B_6_ Contents (mg)		Almost Never		Twice in a Week		Once in Two Days		Almost Every Day		*p* Value
Cooked rice	35	0.02	CON	0	(0%)	1	(3%)	3	(9%)	31	(89%)	0.236
			DEP	0	(0%)	3	(9%)	7	(20%)	25	(71%)	
Bread	95	0.03	CON	7	(20%)	15	(43%)	5	(14%)	8	(23%)	0.461
			DEP	8	(23%)	9	(26%)	7	(20%)	11	(31%)	
Soybean curd (Tofu)	98	0.05	CON	3	(9%)	14	(40%)	13	(37%)	5	(14%)	0.930
			DEP	3	(9%)	12	(34%)	16	(46%)	4	(11%)	
Soy milk	53	0.06	CON	30	(86%)	2	(6%)	2	(6%)	1	(3%)	0.536
			DEP	25	(71%)	3	(9%)	4	(11%)	3	(9%)	
Fermented soybeans	240	0.24	CON	23	(66%)	9	(26%)	1	(3%)	2	(6%)	0.386
			DEP	17	(49%)	11	(31%)	4	(11%)	3	(9%)	
Nuts	360	0.36	CON	29	(83%)	5	(14%)	1	(3%)	0	(0%)	0.355
			DEP	23	(66%)	10	(29%)	2	(6%)	0	(0%)	
Chestnuts	57	0.37	CON	33	(94%)	2	(6%)	0	(0%)	0	(0%)	0.151
			DEP	28	(80%)	6	(17%)	1	(3%)	0	(0%)	
Spinach	41	0.14	CON	14	(40%)	17	(49%)	4	(11%)	0	(0%)	0.444
			DEP	9	(26%)	21	(60%)	5	(14%)	0	(0%)	
Green soybeans	150	0.15	CON	27	(77%)	7	(20%)	1	(3%)	0	(0%)	1.000
			DEP	26	(74%)	8	(23%)	1	(3%)	0	(0%)	
Green peppers	11	0.19	CON	10	(29%)	16	(46%)	9	(26%)	0	(0%)	0.403
			DEP	14	(40%)	13	(37%)	8	(23%)	0	(0%)	
Avocado	34	0.32	CON	26	(74%)	7	(20%)	2	(6%)	0	(0%)	1.000
			DEP	26	(74%)	6	(17%)	2	(6%)	1	(3%)	
Kiwi fruit	14	0.12	CON	27	(77%)	7	(20%)	1	(3%)	0	(0%)	0.752
			DEP	30	(86%)	4	(11%)	1	(3%)	0	(0%)	
Bananas	10	0.38	CON	19	(54%)	8	(23%)	6	(17%)	2	(6%)	0.747
			DEP	19	(54%)	10	(29%)	3	(9%)	3	(9%)	
Bonito	300	0.76	CON	29	(83%)	4	(11%)	2	(6%)	0	(0%)	**0.006**
			DEP	17	(49%)	14	(40%)	4	(11%)	0	(0%)	
Tuna	300	0.85	CON	26	(74%)	8	(23%)	1	(3%)	0	(0%)	0.104
			DEP	18	(51%)	15	(43%)	2	(6%)	0	(0%)	
Salmon	250	0.64	CON	20	(57%)	14	(40%)	1	(3%)	0	(0%)	0.319
			DEP	21	(60%)	10	(29%)	4	(11%)	0	(0%)	
Prawns	190	0.12	CON	23	(66%)	9	(26%)	3	(9%)	0	(0%)	1.000
			DEP	22	(63%)	9	(26%)	3	(9%)	1	(3%)	
Pacific saury	220	0.51	CON	27	(77%)	7	(20%)	1	(3%)	0	(0%)	**0.022**
			DEP	17	(49%)	17	(49%)	1	(3%)	0	(0%)	
Horse mackerel	220	0.30	CON	30	(86%)	4	(11%)	1	(3%)	0	(0%)	**0.038**
			DEP	21	(60%)	12	(34%)	2	(6%)	0	(0%)	
Mackerel	230	0.59	CON	30	(86%)	4	(11%)	1	(3%)	0	(0%)	**0.001**
			DEP	16	(46%)	16	(46%)	3	(9%)	0	(0%)	
Beef round	240	0.44	CON	14	(40%)	12	(34%)	8	(23%)	1	(3%)	0.108
			DEP	8	(23%)	21	(60%)	6	(17%)	0	(0%)	
Beef liver	290	0.89	CON	34	(97%)	0	(0%)	1	(3%)	0	(0%)	**0.025**
			DEP	29	(83%)	6	(17%)	0	(0%)	0	(0%)	
Pork ham	240	0.37	CON	4	(11%)	14	(40%)	14	(40%)	3	(9%)	0.559
			DEP	2	(6%)	11	(31%)	20	(57%)	2	(6%)	
Chicken breast	230	0.35	CON	5	(14%)	18	(51%)	11	(31%)	1	(3%)	0.735
			DEP	3	(9%)	17	(49%)	12	(34%)	3	(9%)	
Chicken liver	270	0.65	CON	33	(94%)	2	(6%)	0	(0%)	0	(0%)	**0.023**
			DEP	25	(71%)	10	(29%)	0	(0%)	0	(0%)	
Milk	45	0.03	CON	9	(26%)	7	(20%)	5	(14%)	14	(40%)	0.336
			DEP	7	(20%)	10	(29%)	9	(26%)	8	(23%)	
Yoghurt	48	0.04	CON	4	(11%)	16	(46%)	9	(26%)	6	(17%)	0.339
			DEP	9	(26%)	11	(31%)	11	(31%)	4	(11%)	
Cheese	290	0.01	CON	10	(29%)	15	(43%)	7	(20%)	3	(9%)	0.763
			DEP	7	(20%)	14	(40%)	10	(29%)	4	(11%)	
Potato chips	58	0.54	CON	29	(83%)	6	(17%)	0	(0%)	0	(0%)	0.486
			DEP	26	(74%)	7	(20%)	2	(6%)	0	(0%)	
Fried potato	34	0.35	CON	27	(77%)	7	(20%)	1	(3%)	0	(0%)	0.678
			DEP	24	(69%)	9	(26%)	2	(6%)	0	(0%)	

CON: control group and DEP: higher levels of depressive symptoms group. The proportions of four responses: “almost never”, “twice in a week”, “once in two days”, and “almost every day” were compared among the two groups using the chi-square test. If any cell had a frequency < 5, Fisher’s exact test was used. Significant *p* values are in bold type.

**Table 3:** Frequency component of heart rate variability.

	CON		DEP		*t* Value	Cohen’s d	*p* Value
	*n* = 35		*n* = 35				
LF power (ln ms^2^)	5.84	(1.00)	6.28	(1.12)	−1.71	0.37	0.092
HF power (ln ms^2^)	5.48	(1.01)	5.87	(1.08)	−1.49	0.34	0.142
Total power (HF + LF) (ln ms^2^)	6.46	(0.91)	6.88	(1.03)	−1.78	0.40	0.080
LF/HF	2.04	(1.74)	2.19	(1.88)	−0.346	0.04	0.730

Values are presented as means (SD). CON: control group; DEP: higher levels of depressive symptoms group; LF: low-frequency component; and HF: high-frequency component.
